# Relevance of Complement C4 Deposits Localized to Distinct Vascular Compartments in ANCA-Associated Renal Vasculitis

**DOI:** 10.3390/ijms232214325

**Published:** 2022-11-18

**Authors:** Samy Hakroush, Ingmar Alexander Kluge, Eva Baier, Désirée Tampe, Björn Tampe

**Affiliations:** 1 Institute of Pathology, University Medical Center Göttingen, 37075 Göttingen, Germany; 2 SYNLAB Pathology Hannover, SYNLAB Holding Germany, 86156 Augsburg, Germany; 3 Department of Nephrology and Rheumatology, University Medical Center Göttingen, 37075 Göttingen, Germany

**Keywords:** ANCA-associated vasculitis, glomerulonephritis, complement deposition, C4 deposits

## Abstract

Anti-neutrophil cytoplasmic antibody (ANCA)-associated vasculitis (AAV) is a small-vessel vasculitis affecting multiple organ systems, including the kidney. Small vessels in the kidney include small-sized arteries, capillaries, and venules. Intrarenal C4 deposits are now increasingly recognized as a potential marker and pathogenic mechanism of autoantibody-mediated tissue damage in ANCA-associated renal vasculitis. We here describe the relevance of complement C4 deposits localized to distinct vascular compartments in a cohort of biopsy-proven ANCA-associated renal vasculitis. A cohort of 43 biopsy-proven cases of ANCA-associated renal vasculitis with myeloperoxidase (MPO) or proteinase 3 (PR3) seropositivity were retrospectively enrolled in a single-center observational study. Univariate and multivariate regression analysis was performed to identify parameters associated with intrarenal C4 deposits in ANCA-associated renal vasculitis. We here show that C4 deposits localize to distinct vascular compartments in ANCA-associated renal vasculitis, and provide evidence for an association with better short-term survival (*p* = 0.008), implicating that this subgroup had a superior response to remission induction therapy. Second, C4 deposits in interlobular arteries were associated with eosinophilic infiltrates in renal vasculitis with MPO-ANCA seropositivity (*p* = 0.021). In renal vasculitis positive for MPO-ANCA, the absence of C4 deposits in the glomerular tuft was associated with sclerotic class ANCA-associated renal vasculitis (*p* < 0.001), and tubular RBC casts (*p* = 0.024). Fourth, complement C4 in interlobular arteries is associated with tubular atrophy specifically in renal vasculitis with PR3-ANCA seropositivity (*p* = 0.006). Finally, complement C4 deposits in peritubular capillaries associated specifically with hyaline casts in cases positive for PR3-ANCA (*p* = 0.025), implicating a role in tubular injury. Interestingly, C4 deposits were localized to distinct vascular compartments independent of the systemic activation of the complement system, reflected by the consumption of respective serum complement molecules in ANCA-associated renal vasculitis. In summary, we here show that C4 deposits localize to distinct vascular compartments in ANCA-associated renal vasculitis, and provide evidence for an association with survival and distinct histopathological lesions. Considering recent advances in AAV therapy with the emergence of new therapeutics that inhibit complement activation, we here provide novel insights into complement C4 as a potential marker to identify patients who may benefit most from these drugs. Thus, our results may contribute to a more personalized treatment approach of AAV depending on the relevance of distinct intrarenal complement deposits.

## 1. Introduction

Anti-neutrophil cytoplasmic antibody (ANCA)-associated vasculitis (AAV) is a small-vessel vasculitis affecting multiple organ systems, including the kidney [1,2,3]. Small vessels in the kidney include small-sized arteries, capillaries, and venules. Disease manifestations in the kidney are common and usually characterized by pauci-immune glomerulonephritis with only minor, if any, immunoglobulin and complement depositions in the vascular system. On a mechanistic level, pathogenic ANCA autoantibodies activate neutrophils, causing a release of inflammatory cytokines, reactive oxygen species, and lytic enzymes, resulting in inflammation and vascular injury [4]. The importance of intrarenal complement C3 deposits has already been described, while the relevance of complement C4 deposits localized to distinct vascular compartments in ANCA-associated renal vasculitis remains elusive [5,6]. We have recently reported that a minor fraction of patients with ANCA-associated renal vasculitis and with poor outcome experienced isolated complement C4 lowering not captured by serum C3 measurements [7,8]. Importantly, classical complement system activation mediated by autoantibody–antigen recognition directed against host cells has been observed as a mesangiocapillary pattern of glomerular C4 deposits in ANCA-associated renal vasculitis [5]. Complement C4 has an internal thioester, enabling the formation of a stable covalent bond with any free hydrogen group on target cells. Covalently bound complement C4 has a long half-life at the site of complement activation, and is considered as a footprint of autoantibody-mediated tissue injury. Intrarenal C4 deposits are now increasingly recognized as a potential marker and pathogenic mechanism of autoantibody-mediated tissue damage in ANCA-associated renal vasculitis [5]. Therefore, detailed knowledge of distinct complement components contributing to kidney injury could be of relevance to improve current strategies targeting the complement system in ANCA-associated renal vasculitis. We here describe the relevance of complement C4 deposits localized to distinct vascular compartments in a cohort of biopsy-proven ANCA-associated renal vasculitis [9].

## 2. Results

**Figure 1:**
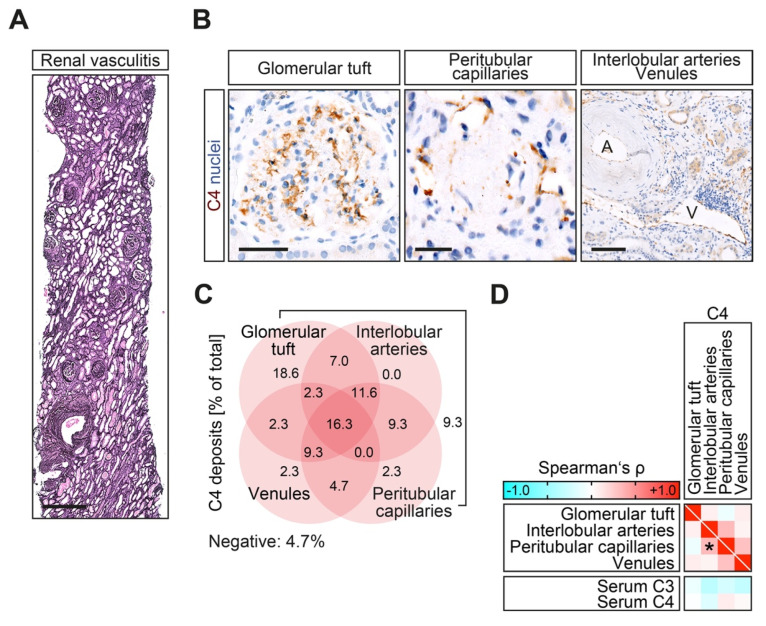
Complement C4 deposits localize to distinct vascular compartments and associate with clinicopathological characteristics in ANCA-associated renal vasculitis. (**A**,**B**) Representative photomicrographs of silver stain and complement C4 with hematoxylin counterstain in the glomerular tuft with typical mesangiocapillary pattern, peritubular capillaries, interlobular arteries (**A**), and venules (V), scale bars from left to right: 500 μm, 25 μm, 50 μm, and 100 μm. (**C**) The presence of complement C4 deposits in the glomerular tuft, interlobular arteries, peritubular capillaries, and venules is presented as a percentage (%) of the total. (**D**) Association between the localization of C4 deposits and serum levels of C3c and C4 in ANCA-associated renal vasculitis are shown by heatmap reflecting mean values of Spearman’s ρ. The asterisk (*) indicates a significant association in the univariate analysis (*p* < 0.05).

In the total cohort (Table 1), complement C4 deposits localized to either the glomerular tuft, interlobular arteries, peritubular capillaries, or venules were present in 41/43 (95.3%) of ANCA-associated renal vasculitis cases (Figure 1A,B and Table 2). Most C4 deposits were only localized to the glomerular tuft with a typical mesangiocapillary pattern in 8/43 (18.6%) of cases, followed by an overlap with C4 deposits in interlobular arteries, peritubular capillaries, and/or venules with presence in all compartments in 7/43 (16.3%) of cases (Figure 1C and Table 2). The only association was observed between C4 deposits in peritubular capillaries and interlobular arteries (Figure 1D), suggesting that intrarenal C4 deposits localized to distinct vascular compartments are a specific observation and not indicative for systemic complement activation. This is further supported by the fact that there is no association between intrarenal C4 deposits and respective serum complement molecules C3 and C4 (Figure 1D). In summary, we observed the presence of complement C4 deposits in the majority of ANCA-associated renal vasculitis cases. Furthermore, C4 deposits were localized to distinct vascular compartments independent of the systemic activation of the complement system.

**Figure 2:**
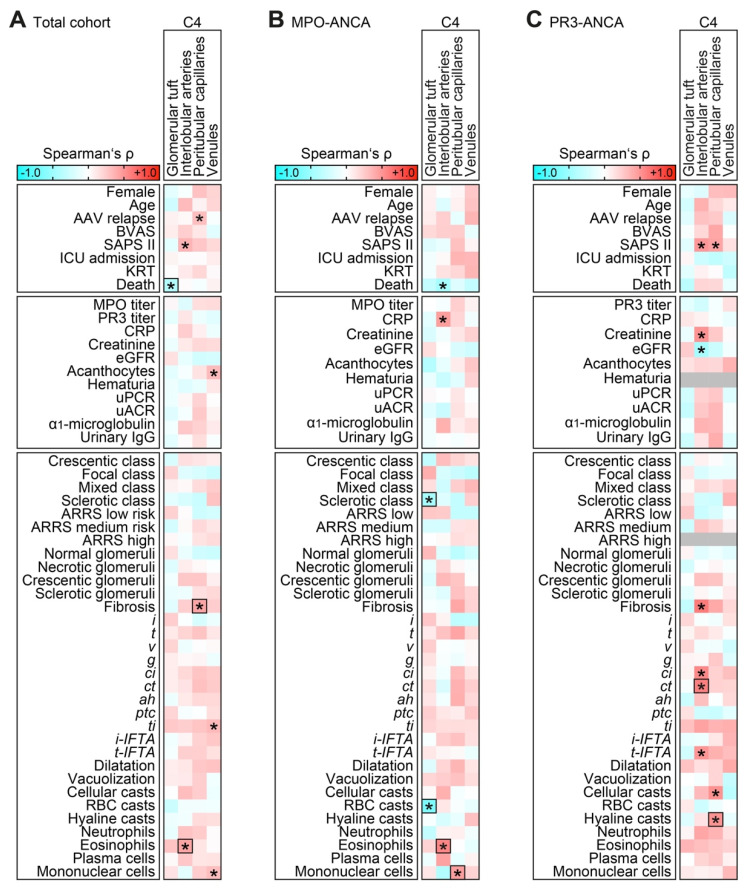
Complement C4 deposits associate with clinicopathological characteristics in ANCA-associated renal vasculitis. (**A**–**C**) Association between C4 deposits localized to the glomerular tuft, interlobular arteries, peritubular capillaries, and venules; clinicopathological characteristics are shown by heatmap reflecting mean values of Spearman’s ρ. Asterisks (*) indicate significant associations in the univariate analysis (*p* < 0.05), rectangle boxes indicate significant associations in the stepwise regression analysis (*p* < 0.05). The grey line for hematuria within the heatmap reflects no data analysis because the respective parameter was present in all cases; the grey line for ARRS high risk indicates that the respective parameter was absent in all cases.

We next analyzed C4 deposits localized to distinct vascular compartments in association with clinicopathological characteristics in ANCA-associated renal vasculitis. Via univariate and stepwise regression analysis, we discovered that C4 deposits localized to the glomerular tuft were associated with better short-term survival within 30 days after admission (β = −0.401, *p* = 0.008), and did not correlate with any histopathological lesion (Figure 2A). Moreover, the presence of C4 deposits in interlobular arteries correlated with eosinophilic inflammation in the kidney (β = 0.384, *p* = 0.013). Finally, C4 deposits in peritubular capillaries was associated with interstitial fibrosis (β = 0.393, *p* = 0.009, Figure 2A). We next analyzed distinct associations within ANCA subtypes. In renal vasculitis positive for MPO-ANCA, the absence of C4 deposits in the glomerular tuft was associated with sclerotic class ANCA-associated renal vasculitis (β = −0.356, *p* = 0.024) and tubular RBC casts (β = −0.657, *p* < 0.001, Figure 2B). C4 deposits in interlobular arteries correlated again with eosinophilic infiltrates in MPO-ANCA (β = 0.512, *p* = 0.021, Figure 2B). Moreover, C4 deposits in interlobular arteries were associated with tubular atrophy (*ct*) particularly in renal vasculitis with PR3-ANCA seropositivity (β = 0.588, *p* = 0.006, Figure 2C). Finally, C4 deposits in peritubular capillaries correlated specifically with hyaline casts in renal vasculitis positive for PR3-ANCA (β = 0.501, *p* = 0.025, Figure 2C).

**Figure 3:**
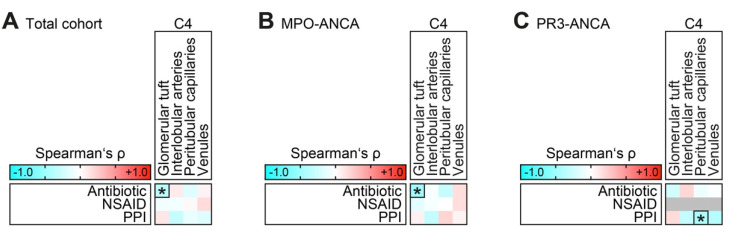
Association between intrarenal C4 deposits and co-medication in ANCA-associated renal vasculitis. (**A**–**C**) Association between antibiotic treatment of infectious complications and C4 deposits localized to the glomerular tuft, interlobular arteries, peritubular capillaries, and venules are shown by heatmap reflecting mean values of Spearman’s ρ. Asterisks (*) indicate significant associations in the univariate analysis (*p* < 0.05), rectangle boxes indicate significant associations in the stepwise regression analysis (*p* < 0.05). The grey line for NSAID within the heatmap reflects no data analysis because the respective parameter was absent in all cases.

We finally assessed the impact of co-medications with an antibiotic, non-steroidal, anti-inflammatory drug (NSAID), or proton pump inhibitor (PPI) on complement C4 deposits in ANCA-associated renal vasculitis. The presence of C4 deposits in the glomerular tuft inversely correlated with antibiotic treatment (β = −0.354, *p* = 0.020, Figure 3A), specifically in renal vasculitis with MPO-ANCA seropositivity (β = −0.495, *p* = 0.022, Figure 3B). However, there was no significant association between antibiotic treatment and short-term survival in ANCA-associated renal vasculitis (β = 0.202, *p* = 0.195). Finally, there was an inverse association between C4 deposits in peritubular capillaries and PPI use, specifically in renal vasculitis with PR3-ANCA seropositivity (β = −0.437, *p* = 0.042, Figure 3C). In summary, we observed an association between complement C4 deposits localized to distinct vascular compartments and clinicopathological characteristics in ANCA-associated renal vasculitis differing between ANCA subtypes.

## 3. Discussion

To our knowledge, this is the first report of a systematic analysis of intrarenal complement C4 deposits localized to distinct vascular compartments in ANCA-associated renal vasculitis. As previously observed, C4 deposits present in the vast majority were predominantly localized to the glomerular tuft and did not correlate with any histopathological lesion [5]. However, C4 deposits were also present in interlobular arteries, peritubular capillaries, and venules in a considerable subset of ANCA-associated renal vasculitis cases. Interestingly, we identified that the relevance of intrarenal complement C4 deposits in ANCA-associated renal vasculitis differed between ANCA subtypes. AAV has long been considered a group of disorders that represent a single disease spectrum with common pathomechanisms. Genome-wide association studies have challenged this concept, with strong genetic associations linked to the antigenic specificity of ANCA, rather than the clinical phenotype [10]. Specifically, MPO-ANCA has been associated with a single nucleotide polymorphism in human leukocyte antigen (HLA)-DQ and the PR3-ANCA with HLA-DP, α1-antitrypsin, and PR3-associated genes.

Our analysis of clinicopathological characteristics identified the following associations: First, the presence of C4 deposits in the glomerular tuft correlated with better short-term survival. Infectious complications have been shown to trigger MPO-ANCA and vasculitis that might impact outcome and survival [11]. However, we did not observe a direct association between infectious complications reflected by antibiotic treatment and short-term survival. Therefore, these results may implicate that this subgroup had a superior response to remission induction therapy. Second, C4 deposits in interlobular arteries were associated with eosinophilic infiltrates in renal vasculitis with MPO-ANCA seropositivity. Interestingly, membrane receptors for complement components, including C4, have already been shown to be present on eosinophils [12]. Third, the absence of C4 deposits in the glomerular tuft was related to the sclerotic class and RBC casts in renal vasculitis with MPO-ANCA seropositivity. This finding is in line with previous observations that eosinophils express receptors for various complement proteins, known to promote eosinophil recruitment, extravasation, and activation [13]. Fourth, complement C4 in interlobular arteries is associated with tubular atrophy specifically in renal vasculitis with PR3-ANCA seropositivity. This is especially relevant since renal arteritis and tubular atrophy have independently been identified to correlate with a poor long-term renal outcome [14,15]. Finally, complement C4 deposits in peritubular capillaries associated specifically with hyaline casts in cases positive for PR3-ANCA, implicating a role in tubular injury. Importantly, C4 deposits localized to any vascular compartments was independent of systemic complement system activation, as reflected by the consumption of respective serum complement molecules in ANCA-associated renal vasculitis. These observations further support the distinct roles of complement components and the concept of intrarenal complement synthesis contributing to complement deposition and kidney injury in ANCA-associated renal vasculitis [6]. This is in line with independent reports that hypocomplementemia is uncommon in ANCA-associated renal vasculitis and only observed in a minor fraction of cases [7,8,16]. Complement C4 is increasingly recognized as a potential marker of autoantibody-mediated tissue damage in AAV [5]. Considering recent advances in AAV therapy with the emergence of new therapeutics that inhibit complement activation, we here provide novel insights into complement C4 as a potential marker to identify patients who may benefit most from these drugs [17]. Particularly, two complement system inhibitors are currently in clinical development for AAV, namely, the oral C5a receptor (C5aR) inhibitor avacopan and the monoclonal C5a antibody IFX-1 [18]. Safety and efficacy with steroid-sparing effects of avacopan in patients with GPA/MPA were shown in Phase II and III clinical trials [17,19]. Furthermore, IFX-1 has entered Phase II development [18]. Thus, our results may contribute to a more personalized treatment approach of AAV depending on the relevance of distinct intrarenal complement deposits.

This study has limitations. Our conclusions are based on a relatively small number of cases and a retrospective study design in a single center, and with no independent validation of our findings. Therefore, larger prospective studies to interrogate the generalizability of our findings is required.

## 4. Materials and Methods

### 4.1. Study Population

A total number of 43 kidney biopsies with ANCA-associated renal vasculitis at the University Medical Center Göttingen were retrospectively included between 2015 and 2020, the patient cohort has previously been described [9,20,21,22,23]. While no formal approval was required for the use of routine clinical data, a favorable ethical opinion was granted by the local ethics committee (no. 22/2/14 and 28/09/17). At admission, the Birmingham vasculitis activity score (BVAS) version 3 was assessed [24]. The simplified acute physiology score (SAPS) II was calculated according to the published guidelines [25]. Medical records were used to obtain data on age, sex, duration of disease onset before admission, and laboratory results. The estimated glomerular filtration rate (eGFR) was calculated using the chronic kidney disease epidemiology collaboration (CKD-EPI) equation [26]. Requirement of intensive care treatment was defined at admission to the intensive care unit (ICU) or intermediate care unit (IMC); all patients required critical care treatment for >24 h. When required, renal replacement therapy (KRT) was performed intermittently in all cases. Indications for KRT included severe electrolyte and acid–base abnormalities, volume overload, and encephalopathy. At the time of kidney biopsy, all patients received glucocorticoids (GCs), and further remission induction therapy was initiated thereafter based on histopathological confirmation of ANCA-associated renal vasculitis. GCs were administered either as intravenous pulse therapy or orally with a tapering schedule. Short-term survival was defined within 30 days after admission.

### 4.2. Renal Histopathology

A renal pathologist evaluated the kidney biopsies and was blinded to clinical data. Within a kidney biopsy, each glomerulus was scored separately for the presence of necrosis, crescents, and global sclerosis. Based on these scorings, histopathological subgrouping according to Berden et al. into focal, crescentic, mixed, or sclerotic classes was performed [27]. Furthermore, the ANCA renal risk score (ARRS), according to Brix et al., into low, medium, or high risk, was calculated [28]. The total renal chronicity score, including global/segmental glomerular sclerosis (score 0, <10%; 1, 10–25%; 2, 26–50%; 3, >50%), interstitial fibrosis (score 0, <10%; 1, 10–25%; 2, 26–50%; 3, >50%), tubular atrophy (score 0, <10%; 1, 10–25%; 2, 26–50%; 3, >50%), and arteriosclerosis (score 0, intimal thickening < thickness of media; 1, intimal thickening ≥ thickness of media) was evaluated as previously described [29]. Kidney biopsies were also evaluated analogously to the Banff scoring system for allograft pathology, as described previously [30]. In brief, Banff score lesions include interstitial inflammation (*i*), tubulitis (*t*), arteritis (*v*), glomerulitis (*g*), interstitial fibrosis (*ci*), tubular atrophy (*ct*), arteriolar hyalinosis (*ah*), peritubular capillaritis (*ptc*), total inflammation (*ti*), inflammation in areas of IFTA (*i-IFTA*), and tubulitis in areas of IFTA (*t-IFTA*) [30]. Systematic histological scoring of tubular injury lesions was evaluated as previously described [31,32]. In brief, epithelial simplification and tubular dilation, non-isometric cell vacuolization, cellular, red blood cell (RBC), and hyaline casts were given a score ranging from 0 to 4 as a percentage of the total affected cortical area of the biopsy (score 0, <1%; 1, ≥1–10%; 2, ≥10–25%; 3, ≥25–50%; 4, >50%). In addition, infiltrates of neutrophils, eosinophils, plasma cells, and mononucleated cells (macrophages, lymphocytes) were quantified as a fraction of the area of total cortical inflammation. The total cortical inflammation area, including areas of interstitial fibrosis and tubular atrophy, and the subcapsular and perivascular cortex, including nodular infiltrates, were considered as previously described [33].

### 4.3. ANCA Autoantibody and Complement Measurements

MPO-ANCA (reference range, <3.5 IU/mL) and PR3-ANCA autoantibodies (reference range, <2 IU/mL) were measured by immunoassay (ImmunoCAP 250, Thermo Fisher Scientific, Waltham, MA, USA). Plasma concentrations of human complement components C3c (9D9621, Abbott, Chicago, IL, USA) and C4 (9D9721, Abbott, Chicago, IL, USA) were determined by turbidimetric measurements on the ARCHITECT-C module. The reference plasma concentration range for serum C3 was defined between 0.82–1.93 g/L and for C4 between 0.15–0.57 g/L.

### 4.4. C4d Immunohistochemistry

Formalin-fixed, paraffin-embedded kidney sections were deparaffinized in xylene and rehydrated in ethanol containing distilled water. Tissue sections were stained using antibodies against C4d (1:50, 503–17344, Zytomed, Berlin, Germany), and labeling was performed using a Novolink^TM^ Polymer Detection System (Leica Biosystems, Wetzlar, Germany) according to the manufacturer’s protocol. Nuclear counterstaining was performed using Mayer’s hematoxylin solution (Sigma, St. Louis, MO, USA). Kindey biopsies were evaluated for the presence/absence of C4d deposits in the glomerular tuft, interlobular arteries, peritubular capillaries, and venules.

### 4.5. Statistical Methods

Variables were tested for normal distribution using the Shapiro–Wilk test. Statistical comparisons were not formally powered or prespecified. Continuous and ordinal variables are presented as mean ± SD, and categorical variables as percentages of the total. Spearman’s correlation was performed to assess the correlation between clinical, laboratory, and histopathological parameters, and heatmaps reflecting the mean values of Spearman’s ρ are shown. Data analyses were performed with GraphPad Prism (version 8.4.3 for macOS, GraphPad Software, San Diego, CA, USA). Stepwise multiple regression analyses were performed using IBM SPSS Statistics (version 27 for MacOS, IBM Corporation, Armonk, NY, USA); rectangle boxes indicate statistically significant correlations. We retained covariates significantly associated with C4 deposits in a multivariable regression model, limiting the model covariates to avoid model overfitting. A probability (*p*) value of < 0.05 was considered statistically significant.

## 5. Conclusions

In summary, we here show that C4 deposits localize to distinct vascular compartments in ANCA-associated renal vasculitis, and provide evidence for an association with survival and distinct histopathological lesions. Considering recent advances in AAV therapy with the emergence of new therapeutics that inhibit complement activation, we here provide novel insights into complement C4 as a potential marker to identify patients who may benefit most from these drugs. Thus, our results may contribute to a more personalized treatment approach of AAV depending on the relevance of distinct intrarenal complement deposits.

## Figures and Tables

**Table 1:** Clinical and laboratory parameters of the total cohort of ANCA-associated renal vasculitis.

Parameter	Value
Female sex—no. (%)	16 (37.2)
Age—years	62.1 ± 15
ANCA subtype MPO/PR3—no. (%)	21/22 (48.8/51.2)
AAV relapse—no. (%)	6 (14)
BVAS—points	17.6 ± 4.3
SAPS II—points	25.7 ± 9.7
CRP—mg/L	78.9 ± 77.6
C3c—g/L	1.21 ± 0.31
C4—g/L	0.26 ± 0.08
Serum creatinine—mg/dL	3.6 ± 2.6
eGFR—mL/min/1.73 m^2^	33.8 ± 32.3
Hematuria—no. (%)	42 (97.7)
uPCR—mg/g	1651 ± 2096
uACR—mg/g	851.8 ± 1349
α_1_-microglobulin—mg/g	116.9 ± 117.5
IgG—mg/g	118 ± 174.5

Abbreviations: AAV, ANCA-associated vasculitis; BVAS, Birmingham vasculitis activity score; CRP, C-reactive protein; eGFR, estimated glomerular filtration rate (CKD-EPI); IgG, immunoglobulin G; MPO, myeloperoxidase; no., number; PR3, proteinase 3; SAPS II, simplified acute physiology score; uACR, urinary albumin-to-creatinine ratio; uPCR, urinary protein-to-creatinine ratio.

**Table 2:** Frequency of complement C4 deposits in the total cohort of ANCA-associated renal vasculitis.

Complement C4 Deposits	Value
Any C4 deposits—no. (%)	41 (95.3)
Glomerular tuft—no. (%)	33 (76.7)
Interlobular arteries—no. (%)	20 (46.5)
Peritubular capillaries—no. (%)	27 (62.8)
Venules—no. (%)	16 (37.2)

Abbreviations: ANCA, anti-neutrophil cytoplasmic antibodies; no., number.

## Data Availability

The original contributions presented in the study are included in the article, further data and material are available from the corresponding author upon reasonable request.
